# Reelin Rescues Behavioral, Electrophysiological, and Molecular Metrics of a Chronic Stress Phenotype in a Similar Manner to Ketamine

**DOI:** 10.1523/ENEURO.0106-23.2023

**Published:** 2023-08-09

**Authors:** Jenessa N. Johnston, Josh Allen, Irene Shkolnikov, Carla L. Sanchez-Lafuente, Brady S. Reive, Kaylene Scheil, Stanley Liang, Brian R. Christie, Lisa E. Kalynchuk, Hector J. Caruncho

**Affiliations:** Division of Medical Sciences, University of Victoria, Victoria, British Columbia V8P 5C2, Canada

**Keywords:** corticosterone, depression, ketamine, long-term potentiation, reelin, synaptic plasticity

## Abstract

Over the past decade, ketamine, an NMDA receptor antagonist, has demonstrated fast-acting antidepressant effects previously unseen with monoaminergic-based therapeutics. Concerns regarding psychotomimetic effects limit the use of ketamine for certain patient populations. Reelin, an extracellular matrix glycoprotein, has shown promise as a putative fast-acting antidepressant in a model of chronic stress. However, research has not yet demonstrated the changes that occur rapidly after peripheral reelin administration. To address this key gap in knowledge, male Long–Evans rats underwent a chronic corticosterone (CORT; or vehicle) paradigm (40 mg/kg, 21 d). On day 21, rats were then administered an acute dose of ketamine (10 mg/kg, i.p.), reelin (3 µg, i.v.), or vehicle. Twenty-four hours after administration, rats underwent behavioral or *in vivo* electrophysiological testing before killing. Immunohistochemistry was used to confirm changes in hippocampal reelin immunoreactivity. Lastly, the hippocampus was microdissected from fresh tissue to ascertain whole cell and synaptic-specific changes in protein expression through Western blotting. Chronic corticosterone induced a chronic stress phenotype in the forced swim test and sucrose preference test (SPT). Both reelin and ketamine rescued immobility and swimming, however reelin alone rescued latency to immobility. *In vivo* electrophysiology revealed decreases in hippocampal long-term potentiation (LTP) after chronic stress which was increased significantly by both ketamine and reelin. Reelin immunoreactivity in the dentate gyrus paralleled the behavioral and electrophysiological findings, but no significant changes were observed in synaptic-level protein expression. This exploratory research supports the putative rapid-acting antidepressant effects of an acute dose of reelin across behavioral, electrophysiological, and molecular measures.

## Significance Statement

The discovery of ketamine as a rapid-acting antidepressant has been the first major neuropharmacological discovery in decades. However, adverse side effects limit the widespread use of ketamine to certain patient populations. Reelin, a protein that is downregulated in patients with depression, appears to work on similar pathways to the antidepressant effects of ketamine. This study demonstrates that an acute peripheral dose of reelin may rescue certain behavioral, electrophysiological, and molecular deficits in a chronic stress phenotype. The upregulation of reelin expression may provide a novel therapeutic target that could be useful in many patient populations.

## Introduction

Major depressive disorder (MDD) is the most prevalent neuropsychiatric disorder worldwide, affecting around 16% of people throughout their lifetime (Friedrich, 2017). Traditional monoaminergic-based antidepressants, such as selective serotonin reuptake inhibitors (SSRIs), have failed to fully address MDD, with around 50–60% of patients failing to respond to first-line treatment (Rush et al., 2006).

Ketamine, an NMDA receptor (NMDAR) antagonist, has rapid-acting antidepressant effects previously unseen (Berman et al., 2000; Zarate et al., 2006; Murrough et al., 2013; Dai et al., 2022). While there is significant debate on the exact mechanism of ketamine’s antidepressant actions, they are generally thought to be mediated through an increase in cortical and hippocampal excitatory transmission (Abdallah et al., 2018; Duman et al., 2019; Gilbert and Zarate, 2020). Despite ketamine’s success, there are still concerns regarding long-term use which may limit its widespread application to certain patient populations (Short et al., 2018; Alnefeesi et al., 2022).

A glutamate “surge” has been consistently observed after ketamine administration, leading to an increase in AMPA receptor (AMPAR) transmission and subsequent synaptic-level signaling pathways which induce increases in mechanistic target of rapamycin complex 1 (mTORC1) activity, postsynaptic density (PSD)-95 expression, and surface insertion of GluA1 AMPAR subunits (Li et al., 2010; Zanos et al., 2016, 2018a,b).

Reelin, an extracellular matrix protein released by GABAergic interneurons in adulthood, is downregulated in the hippocampus of patients with depression (Fatemi et al., 2000, 2001). In addition, reelin is known to regulate dendritogenesis and synaptogenesis (Lee and D’Arcangelo, 2016; Wasser and Herz, 2017). Heterozygous reeler mice (HRM), with ∼40–60% less endogenous reelin, are more vulnerable to the depressogenic effects of chronic corticosterone (CORT) administration, demonstrating dose-dependent behavioral changes and decreases in markers of neurogenesis (Lussier et al., 2011). Interestingly, decreases in molecules such as PSD-95, activity-regulated cytoskeletal protein (Arc), and dendritic complexity have been observed in HRM (Dong et al., 2003; Ventruti et al., 2011).

The hippocampus is extremely susceptible to stress-induced impairments because of a high prevalence of glucocorticoid receptors in limbic regions (Herman et al., 2005). One of the main deficits observed is a decrease in hippocampal long-term potentiation (LTP), associated with a loss of learning and memory that is often observed in depression and depressive-like phenotypes (Kizilbash et al., 2002; Lynch, 2004). Ketamine has been shown to increase hippocampal LTP, learning, and memory in a fast-acting manner (Gilbert and Zarate, 2020; Sumner et al., 2020a,b). Reelin application has been able to rescue cognition, synaptic plasticity, and dendritic spinogenesis (Rogers et al., 2011, 2013; Hethorn et al., 2015), but no research to our knowledge has assessed the effect of peripheral reelin administration on LTP after chronic stress.

In a chronic stress model, the decrease of reelin expression parallels the progressive development of depressive-like behavior (Lussier et al., 2013; Lebedeva et al., 2020; Sánchez-Lafuente et al., 2022). Previous research from our laboratory has demonstrated that reelin and ketamine have parallel effects on synaptoneurosomes (SNPs; isolated presynaptic and postsynaptic compartments) after *in vitro* treatment, dose-dependently increasing expression of PSD-95, mTORC1, and p-mTORC1 (Brymer et al., 2020; Johnston et al., 2020). However, no research has demonstrated changes after *in vivo* administration of reelin.

Administration of both traditional (chronic imipramine administration) and nontraditional (etanercept, a TNF-α inhibitor) antidepressants can rescue hippocampal reelin expression (Fenton et al., 2015; Brymer et al., 2018). Previous research from our laboratory has demonstrated that both repeated and acute intrahippocampal infusions of recombinant reelin (1 µg) were able to rescue stress-induced deficits in object-location memory, forced-swim test immobility, and hippocampal AMPAR, NMDAR, and GABA_A_R expression in the hippocampus. (Brymer et al., 2020). In addition, peripheral administration of reelin through the lateral tail vein demonstrated a parallel rescue of behavioral and biological chronic stress phenotype (Allen, 2022). The actions of reelin in chronic stress models may be activated through similar AMPAR-mediated downstream cellular signaling pathways to ketamine, as blocking AMPAR signaling with CNQX prevents reelin’s ability to rescue a chronic stress phenotype (Brymer et al., 2020). Additionally, blockade of reelin’s receptor apolipoprotein E receptor 2 and related downstream Src family signaling kinases prevented ketamine’s actions in a depressive-like phenotype (Kim et al., 2021).

The aim of the present study is to provide an exploratory look at the behavioral, electrophysiological, and molecular effects of reelin in parallel to ketamine, to support reelin’s putative actions in rescuing a chronic stress phenotype within 24 h.

## Materials and Methods

### Animal husbandry

Male Long–Evans rats (*n* = 72) were purchased from Charles River Laboratories aged six weeks weighing between 200 and 250 g. Rats were housed individually with access to food and water *ad libitum* in clear polypropylene cages with a wooden chew cube and red hut. The colony was temperature-controlled at 21°C and maintained on a 12/12 h light/dark cycle that turned lights on at 7 A.M. Purina rat chow was maintained daily, and bedding was changed once per week.

### Experimental procedures

All procedures were conducted in accordance with the Canadian Council on Animal Care and approved by the University of Victoria Animal Ethics Board. Rats were given one week of habituation to the facility before one week of daily handling. After weight was measured, animals were divided into 21 d of daily vehicle (NaCl, 0.9%, 2% polysorbate-80) or CORT (40 mg/kg suspended in vehicle, Steraloids). Rats were weighed daily to accurately administer injections at 1 ml/kg between 8 and 11 A.M.

**Figure 1.**
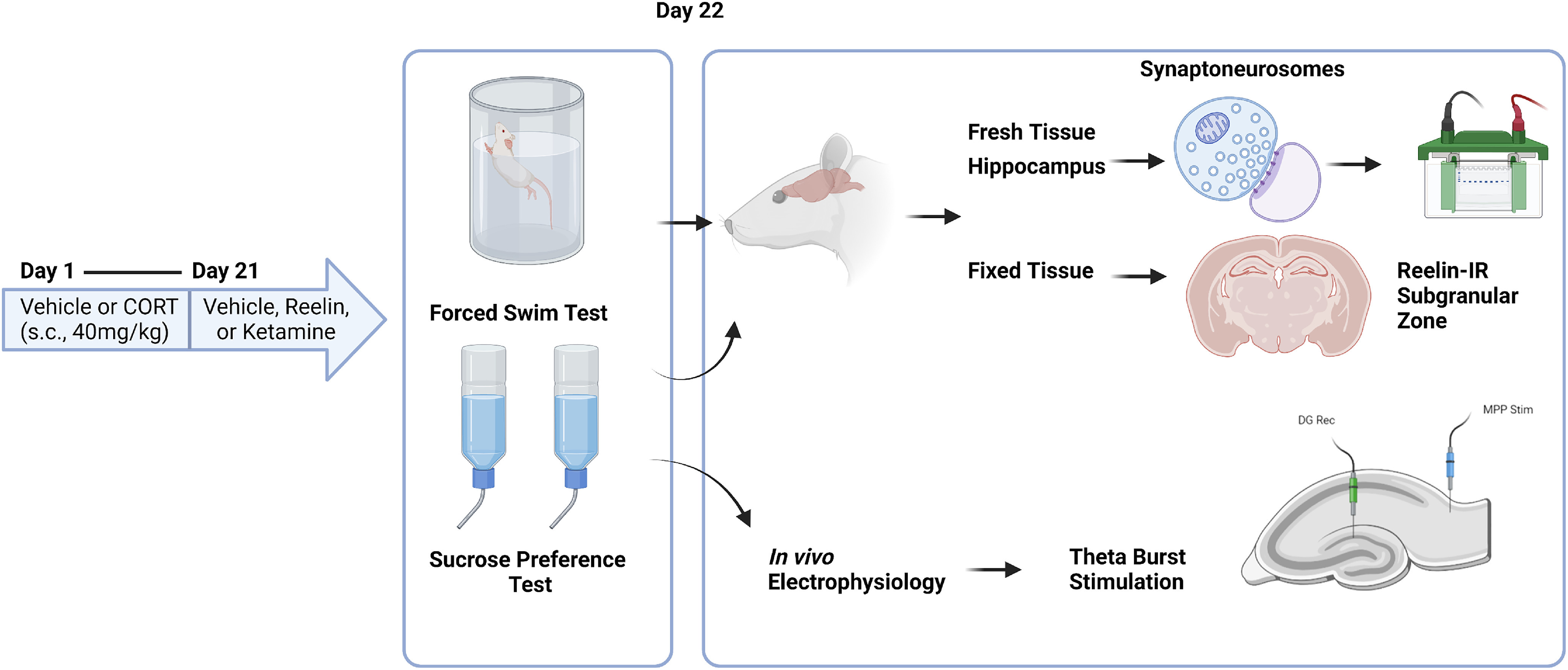
Experimental timeline and analyses. Male Long–Evans rats received daily injections of CORT (40 mg/kg) or vehicle (saline) for 21 d (subcutaneously). On the 21st day, they were administered an injection of reelin (3 µg, i.v.), ketamine (10 mg/kg, i.p.), or vehicle (PBS). Twenty-four hours later, they underwent behavioral testing and killing for fresh or fixed tissue. The animals who underwent *in vivo* electrophysiology underwent a θ burst stimulation protocol to induce LTP, which was recorded from the dentate gyrus. s.c., subcutaneous; MPP, medial perforant path; DG, dentate gyrus.

On day 21, rats were administered an acute dose of vehicle (0.1 м PBS; pH 7.4), recombinant reelin (3 µg, intravenously, suspended in 0.5 ml vehicle), or ketamine hydrochloride (10 mg/kg, i.p., injected at 1 ml/kg in vehicle). The recombinant reelin (3820-MR-025-CF, R&D Systems) used is composed of reelin repeats 3–6 and dosed based on previous research from our lab showing that 3 µg is the most effective dose to rescue depressive-like behavior (Allen et al., 2022). An overview of all experimental procedures can be found in Figure 1.

### Forced swim test

Animals who did not undergo *in vivo* electrophysiology (*n* = 48) were administered a modified 1-d protocol of the forced swim test (FST), which has been previously described (Allen, 2022). A 1-d protocol controls for potential memory-based confounders that may be present in a more traditional 2-d protocol (Porsolt et al., 1978). Rats were placed in a Plexiglas swim tank (25 cm wide × 25 cm long × 60 cm high, filled to 30 cm) for 10 min. Water temperature was held at 27 ± 2°C. Time spent swimming, climbing, immobile, and latency to immobility were all quantified through manual scoring.

### Sucrose preference test

All animals (*n* = 72) underwent the sucrose preference test (SPT), which is a commonly used indicator of anhedonia in animal models. On day 19, rats were habituated to two bottles of water accessible from their cage. Subsequently, they were habituated to two bottles of a sucrose solution (1%) to assess baseline drinking patterns. On the day of the test (day 21), rats were given free access to one bottle of water and one bottle of the sucrose solution for 24 h. Bottles were switched at 12 h to account for any location preferences, and weighed before and after the test.

### Tissue preparation

#### Perfusions

After behavioral tests on day 22, 24 of the animals were deeply anesthetized with isoflurane (5%) and transcardially perfused using 4% (w/v) paraformaldehyde (PFA) dissolved in 0.1м PB (pH 7.4). Brains were stored in the same PFA solution for 48 h at 4°C before being transferred to a 30% sucrose solution with 0.1% sodium azide. Brains were kept in the sucrose solution for at least 72 h before being sectioned in the coronal plane at 30 µm on a cryostat (CM1850 UV, Leica Biosystems) at −20°C. Coronal sections were stored in a cryoprotectant [30% (w/v) sucrose, 1% (w/v) polyvinylpyrrolidone, and 30% (v/v) ethylene glycol in 0.1 m PBS (pH 7.4)] at −20°C until immunohistochemical analyses.

#### Synaptoneurosomes

Twenty-four animals were deeply anesthetized with isoflurane (5%) and killed by decapitation. The hippocampus was microdissected on ice immediately after killing, then snap frozen in liquid nitrogen. Tissue was stored until use at −80°C. After thawing on ice, hippocampal tissue was homogenized in a Potter–Elvehjem homogenizer containing chilled modified Krebs–Henseleit buffer (mKREBs; in mm: 118.5 NaCl, 4.70 KCl, 1.18, MgCl_2_·6H_2_O, 2.50 CaCl_2_·2H_2_O, 1.18 KH_2_PO_4_, 24.90 NaHCO_3_, 10.00 glucose, pH adjusted to 7.4 using 1.0 N HCl) supplemented with protease and phosphotase inhibitor cocktails (#1860932, #78430, ThermoScientific). A portion of this whole homogenate was stored for later analysis, with the remaining homogenate used to create synaptoneurosomes (SNPs). Briefly, the homogenate was passed through sequential filtrations [100 μm pore nylon filters (NY1H02500; EMD Millipore); 5-μm nitrocellulose Durapore membrane filters (SBLP01300; Millipore)], then centrifuged (1000 × *g*, 15 min at 4°C). The supernatant was discarded, and the SNPs pellet was resuspended in mKREBs buffer for Western blotting analyses. Protein concentrations were determined by a detergent compatible protein assay (#5000112, Bio-Rad).

#### *In vivo* electrophysiology

On day 22, rats (*n* = 24) were weighed and anesthetized deeply with urethane (1.5 g/kg, i.p.). Supplemental doses (0.3 ml) were given as necessary until loss of consciousness as determined through withdrawal reflex and respiratory rate. After rats were unconscious, they were placed on a stereotaxic frame (Kopf Instruments) with body temperature kept steady at 37°C using a regulated homeothermic control unit (Harvard Instruments). Tear gel was first applied before surgical incisions, and then again as needed. An incision was made along the dorsal surface of the skull to expose bregma in accordance with sterile protocols. Two holes were drilled anterior to the bregma for grounding electrode placement (stainless steel, 0.011” coated). Holes were also drilled for the recording (stainless steel, 0.008” coated; 3.5 mm posterior and 2.4 mm lateral to bregma) and stimulating (stainless steel, 0.011 mm coated; 7.4 mm posterior and 3.4 mm lateral to bregma) electrodes.

Baseline recordings were measured using a 0.12-ms pulse at 0.0067 Hz. Input/output (I/O) function was used to determine baseline excitatory transmission. Stable baseline recordings were required for at least 30 min before a θ burst stimulation (TBS) protocol was applied to induce LTP. The TBS consisted of 10 bursts of five pulses (0.25 ms in duration) at 400 Hz with a 200-ms interburst interval, which was repeated four times in 30-s intervals. After induction of LTP, baseline stimulation was continued for 1 h. The slope of the rising phase (10–90%) of field EPSPs (fEPSPs) were recorded. To assess post-tetanic potentiation (PTP), fEPSPs were taken immediately after TBS. LTP was assessed through fEPSP slope from 55 to 60 min after TBS. All data are presented as mean percent change from prestimulation baseline. Confirmation of electrode placement was conducted via Cresyl Violet staining.

#### Immunohistochemistry

Every sixth section of the hippocampus was collected for immunohistochemical analyses of reelin-immunoreactive (IR) cells. After rinses in tris-buffered saline, sections were incubated in sodium citrate (pH 6; 85°C) for antigen retrieval, then blocked for 30 min at room temperature [TBS+ 1% BSA (w/v), 10% Triton X-100 (v/v), 15% normal goat serum (v/v)]. The primary antibody (#MAB5366, mouse anti-reelin, EMD Millipore) was diluted in blocking solution at a concentration of 1:1000 overnight at 4°C. Sections were then treated with 10% hydrogen peroxide (in TBS) before secondary antibody incubation (biotinylated goat anti-mouse IgG, 1:500, Sigma-Aldrich). Lastly, tissue was incubated in an avidin-biotin complex (1:500, Vecta Stain Elite ABC reagent, Vector Labs) then visualized through 0.002% (w/v) DAB (Sigma-Aldrich) and 0.0078% (v/v) H_2_O_2_. Sections were then mounted onto polarized glass slides (Thermofisher Superfrost Plus) and coverslipped with Permount media (Thermofisher Scientific) after dehydration.

Reelin-IR cells were imaged using a Zeiss Axioimager M.2 and counted using an unbiased optical fractionator method (Stereo Investigator, 2022.2.1, MBF Bioscience). The area of interest was traced at 2.5× magnification, with stereological analyses undertaken at 20× magnification. Equations for number estimates have been previously described (Johnston et al., 2020; Allen et al., 2022).

#### SDS-PAGE and Western blotting

Ten micograms of protein from each sample (whole homogenate and SNPs) was electrophoretically resolved in 10% TGX StainFree FastCast Acrylamide Solutions (Bio-Rad) at 200 V for 1 h. After resolution, protein was semi-dry transferred onto 0.2 µm PVDF membranes (#1704272, Bio-Rad) using the Trans-Blot Turbo Transfer System (Bio-Rad). After blocking membranes in 5% (w/v) BSA, primary antibodies were applied at 1:1000 overnight at 4°C. Proteins probed from the hippocampus were mTOR (#2972S, CST), p-mTOR (#2971S, CST), Synapsin I (#6710, CST), PSD-95 (#2507S, CST), GluA1 (#13185S, CST), p-GluA1 (#75574S, CST), p-Erk1/2 (extracellular signal regulator kinase; #9101S, CST), CREB (#4820S, CST), p-CREB (#9198S, CST), and GluN2b (UC Davis). Blots were subsequently washed in tris-buffered saline with 1% (v/v) Tween (TBST) before application of appropriate secondaries (horseradish peroxidase linked goat anti-mouse or goat anti-rabbit) at a 1:5000 concentration. To visualize bands, Luminata Crescendo or Classico was used depending on the abundance of protein (#WBLUR0500 and #WBLUC0500, Millipore Sigma). The G:BOX Chemi XRQ imaging system (Syngene) was used to capture chemiluminescent images, and Fiji (version 2.9.0) was used for quantification of bands. Ponceau staining was used for total protein normalization.

#### Experimental design and statistical analyses

As the purpose of these experiments was primarily exploratory, and four animals/group were used for each separate biological experiment (electrophysiology, immunohistochemistry, Western blotting). The total number of male Long–Evans rats used was 72, and behavior was conducted on all rats that did not undergo electrophysiological analyses (*n* = 48). Power analyses were used based on previous behavioral, immunohistochemistry and Western blotting data conducted in our lab, as well as an initial pilot electrophysiological experiment. The groups were as follows to control for both chronic and acute treatments: Vehicle/Vehicle, Vehicle/Ketamine, Vehicle/Reelin, CORT/Vehicle, CORT/Ketamine, CORT/Reelin. Analyses across all experiments aside from Western blotting, which was analyzed in a three-way ANOVA (tissue type × treatment × condition), were conducted using two-way ANOVAs to assess the effects and differences between condition (chronic vehicle or CORT administration) and treatment group (acute administration of vehicle, reelin, or ketamine). To ensure proper representation of relative protein expression in Western blot analyses, technical duplicates or triplicates were analyzed for every protein from each animal. All statistics were conducted on SPSS (v27, IBM). Tukey’s *post hoc* test was used for further comparisons. All data are presented as mean ± SEM. Statistical significance and effect sizes (η^2^) are reported in Table 1. A summary of findings can be found in Tables 1 and 2.

**Table 1:** Statistical significance table

Category	Measure	*F* test	*F* test *p* value	η^2^	VV vs CV	VV vs VR	VV vs VK	VV vs CR	VV vs CK	CV vs VR	CV vs VK	CV vs CR	CV vs CK
Behavior	FST immobility	*F*_(2,41)_ = 5.184	*p* = 0.0098	0.35	0.0003	ns	ns	ns	ns	0.0001	0.016	0.0049	0.0047
	FST swimming	*F*_(2,41)_ = 6.384	*p* = 0.0039	0.34	0.0002	ns	ns	ns	ns	0.0004	0.025	0.0028	0.0034
	FST climbing	*F*_(2,41)_ = 0.3432	*p* = 0.7115	0.14	ns	ns	ns	ns	ns	ns	ns	ns	ns
	FST latency	*F*_(1,41)_ = 7.670	*p* = 0.0084	0.23	0.017	ns	ns	ns	ns	0.005	0.01	0.0423	ns
	SPT habituation	*F*_(2,42)_ = 0.8359	*p* = 0.4406	0.08	ns	ns	ns	ns	ns	ns	ns	ns	ns
	SPT test	*F*_(2,56)_ = 5.984	*p* = 0.0044	0.28	0.0088	ns	0.0019	ns	ns	0.0045	0.0337	ns	ns
													
Electrophysiology	I/O curve	*F*_(25,143)_ = 0.1007	*p* > 0.9999	0.08	ns	ns	ns	ns	ns	ns	ns	ns	ns
	PTP	*F*_(2,18)_ = 7.179	*p* = 0.0051	0.47	0.0008	0.0415	0.0064	ns	ns	ns	ns	ns	ns
	LTP	*F*_(2,18)_ = 3.924	*p* = 0.0385	0.32	0.0045	ns	ns	ns	ns	ns	ns	0.0342	0.0493
													
Immuno-histochemistry	Reelin in the SGZ	*F*_(2,18)_ = 3.732	*p* = 0.0441	0.33	0.0129	ns	ns	ns	ns	ns	ns	0.0421	0.0443
													
Western blotting	p-mTOR	*F*_(2,84)_ = 0.9520	*p* = 0.3901	1.842	ns	ns	ns	ns	ns	ns	ns	ns	ns
	mTOR	*F*_(2,84)_ = 0.5386	*p* = 0.5855	1.185	ns	ns	ns	ns	ns	ns	ns	ns	ns
	p-mTOR/mTOR	*F*_(2,84)_ = 0.3994	*p* = 0.6720	0.862	ns	ns	ns	ns	ns	ns	ns	ns	ns
	GluN2b	*F*_(2,84)_ = 0.1751	*p* = 0.8397	0.4031	ns	ns	ns	ns	ns	ns	ns	ns	ns
	p-GluA1	*F*_(2,84)_ = 0.06545	*p* = 0.9367	0.142	ns	ns	ns	ns	ns	ns	ns	ns	ns
	GluA1	*F*_(2,84)_ = 0.7681	*p* = 0.4671	1.712	ns	ns	ns	ns	ns	ns	ns	ns	ns
	p-GluA1/GluA1	*F*_(2,84)_ = 0.04725	*p* = 0.9539	0.1036	ns	ns	ns	ns	ns	ns	ns	ns	ns
	PSD-95	*F*_(2,72)_ = 0.004226	*p* = 0.9958	0.01097	ns	ns	ns	ns	ns	ns	ns	ns	ns
	Synapsin I	*F*_(2,108)_ = 0.3786	*p* = 0.6857	0.6402	ns	ns	ns	ns	ns	ns	ns	ns	ns
	p-CREB	*F*_(2,60)_ = 0.5084	*p* = 0.6040	1.613	ns	ns	ns	ns	ns	ns	ns	ns	ns
	CREB	*F*_(2,60)_ = 0.1120	*p* = 0.8942	0.3414	ns	ns	ns	ns	ns	ns	ns	ns	ns
	p-CREB/CREB	*F*_(2,60)_ = 0.01594	*p* = 0.9842	0.05143	ns	ns	ns	ns	ns	ns	ns	ns	ns
	p-ERK	*F*_(2,72)_ = 0.2545	*p* = 0.7760	0.554	ns	ns	ns	ns	ns	ns	ns	ns	ns

Statistical results from two-way ANOVAs. Tukey’s *post hoc* analyses were used if significance was found in the two-way ANOVA. Darkened shading indicates that *post hoc* analyses were not used. CREB, cAMP response element-binding protein; Erk1/2, extracellular signal-regulated kinase; FST, forced swim test; I/O, input/output; LTP, long-term potentiation; mTOR, mechanistic target of rapamycin; NS, not significant; p-, phosphorylated-; PSD-95, postsynaptic density-95; PTP, post-tetanic potentiation; SNP, synaptoneurosome; WH, whole homogenate.

**Table 2:** Summary of findings

Method	Measure	CORT	Reelin	Ketamine
Behavior	FST immobility	↑	↓	↓
	FST swimming	↓	↑	↑
	FST climbing	↔	↔	↔
	FST latency to immobility	↓	↑	↔
	SPT habituation	↔	↔	↔
	SPT test	↓	↔	↔
Electrophysiology	I/O curve	↔	↔	↔
	PTP	↓	↔	↔
	LTP	↓	↑	↑
Immunohistochemistry	Reelin	↓	↑	↑
Western blotting	p-mTOR	↔ (WH and SNP)	↔ (WH and SNP)	↑ (WH) ↔ (SNP)
	mTOR	↔ (WH and SNP)	↔ (WH and SNP)	↔ (WH and SNP)
	p-GluA1	↔ (WH and SNP)	↔ (WH and SNP)	↔ (WH and SNP)
	GluA1	↔ (WH and SNP)	↔ (WH and SNP)	↔ (WH and SNP)
	p-CREB	↔ (WH and SNP)	↔ (WH and SNP)	↔ (WH and SNP)
	CREB	↔ (WH and SNP)	↔ (WH and SNP)	↔ (WH and SNP)
	Synapsin I	↔ (WH and SNP)	↔ (WH and SNP)	↔ (WH and SNP)
	PSD-95	↔ (WH and SNP)	↔ (WH and SNP)	↔ (WH and SNP)
	GluN2b	↔ (WH and SNP)	↔ (WH and SNP)	↔ (WH and SNP)
	p-Erk1/2	↔ (WH and SNP)	↔ (WH and SNP)	↔ (WH and SNP)

CORT, corticosterone; CREB, cAMP response element-binding protein; Erk1/2, extracellular signal-regulated kinase; FST, forced swim test; I/O, input/output; LTP, long-term potentiation; mTOR, mechanistic target of rapamycin; p-, phosphorylated-; PSD-95, postsynaptic density-95; PTP, post-tetanic potentiation; SPT, sucrose preference test; SNP, synaptoneurosome; WH, whole homogenate. ↑, increased; ↓, decreased; ↔, unchanged.

## Results

### Forced swim test

**Figure 2.**
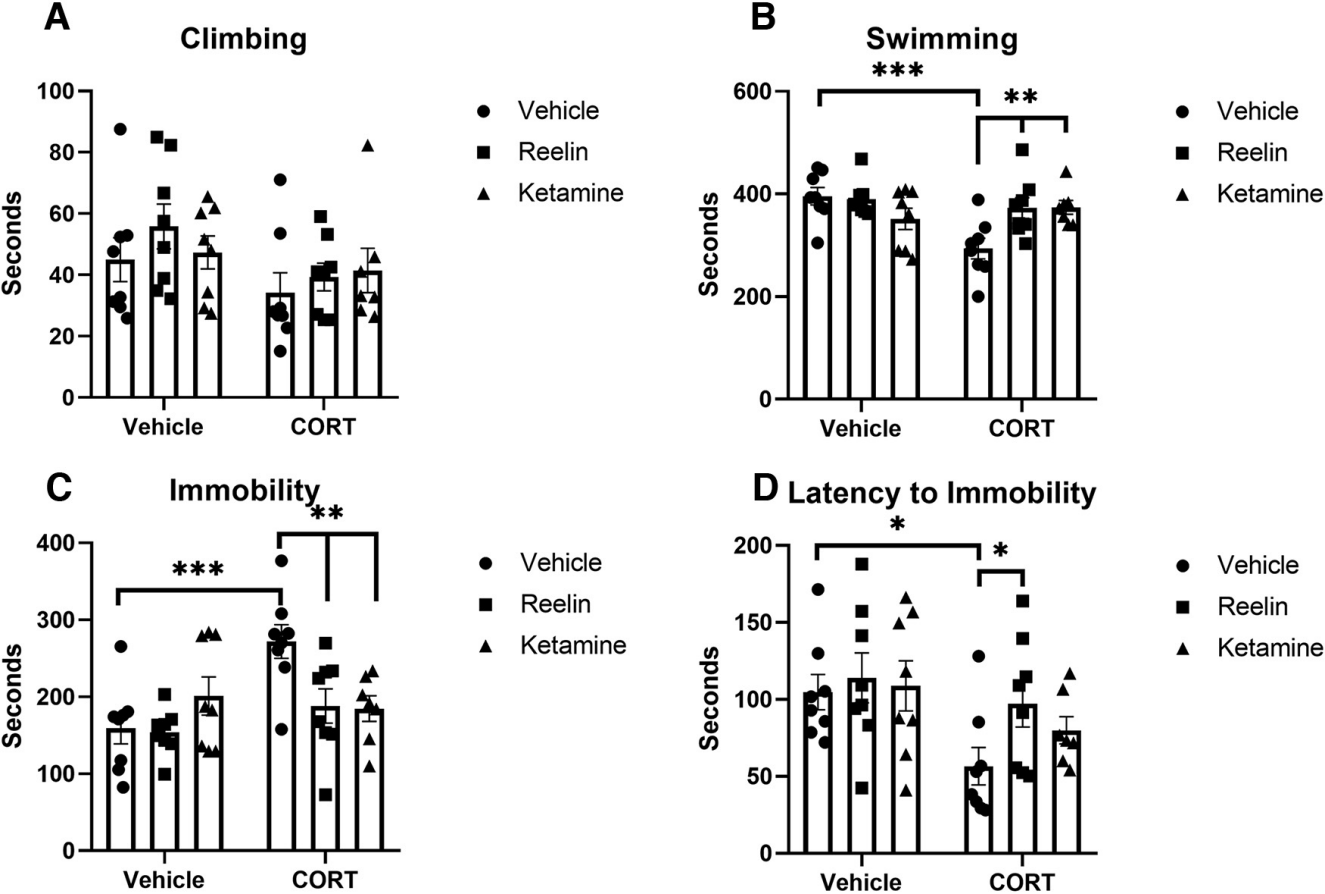
Forced swim test. ***A***, No significant differences were found across groups in FST climbing. ***B***, CORT administration significantly decreased swimming in the FST, which was rescued by both reelin and ketamine. ***C***, CORT administration significantly increased immobility in the FST, which was rescued by both reelin and ketamine. ***D***, Latency to immobility in the FST was decreased by CORT but was rescued only by reelin administration. All data presented as mean ± SEM; *p < 0.05, **p < 0.01, ***p < 0.001.

In the forced swim test, significant differences were found in measures of swimming (*F*_(2,41)_ = 6.384, *p* = 0.0039, η^2^ = 0.34), immobility (*F*_(2,41)_ = 5.184, *p* = 0.0098, η^2^ = 0.35), and latency to immobility (*F*_(1,41)_ = 7.670, *p* = 0.0084, η^2^ = 0.23; Fig. 2*A–D*). *Post hoc* analyses revealed that the CORT/vehicle group spent significantly more time immobile (*p* = 0.0003), an increase that was rescued by reelin (*p* = 0.0049) and ketamine (*p* = 0.0047; Fig. 2*C*). In parallel to this, swimming time was decreased in rats treated with CORT/vehicle (*p* = 0.0002), which was upregulated by reelin (*p* = 0.0028) and ketamine (*p* = 0.0034; Fig. 2*B*). Finally, those in the CORT/vehicle subgroup had significantly shorter latency to immobility times when compared with vehicle-treated animals, which were rescued by reelin (*p* = 0.0423), but not ketamine (Fig. 2*D*). No effects on climbing behavior were found (Fig. 2*A*).

### Sucrose preference test

**Figure 3.**
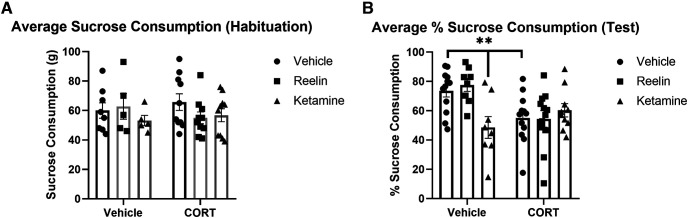
Sucrose consumption after chronic stress, reelin, and ketamine treatment. ***A***, No significant differences were found in sucrose consumption across groups during habituation. ***B***, During the test phase, rats treated with chronic CORT had a significantly decrease consumption of sucrose. In addition, vehicle rats treated with ketamine had significantly decreased sucrose consumption. No significant effect of reelin or ketamine was found. Data expressed as mean ± SEM; **p < 0.01.

Sucrose consumption was measured during the habituation phase (day 20, two bottles of sucrose) and test phase of the SPT (day 21, one bottle of water and one bottle of sucrose) to determine anhedonic-like behavior. During the habituation phase, no significant differences were found between groups (Fig. 3*A*). In the test phase, significant group differences were found (*F*_(2,56)_ = 5.984, *p* = 0.0044, η^2^ = 0.28) in sucrose consumption. The CORT/vehicle and vehicle/ketamine subgroups drank significantly less sucrose than vehicle animals (*p* = 0.009 and *p* = 0.002, respectively), suggesting greater anhedonic-like behavior.

### *In vivo* electrophysiology

#### I/O curve

**Figure 4.**
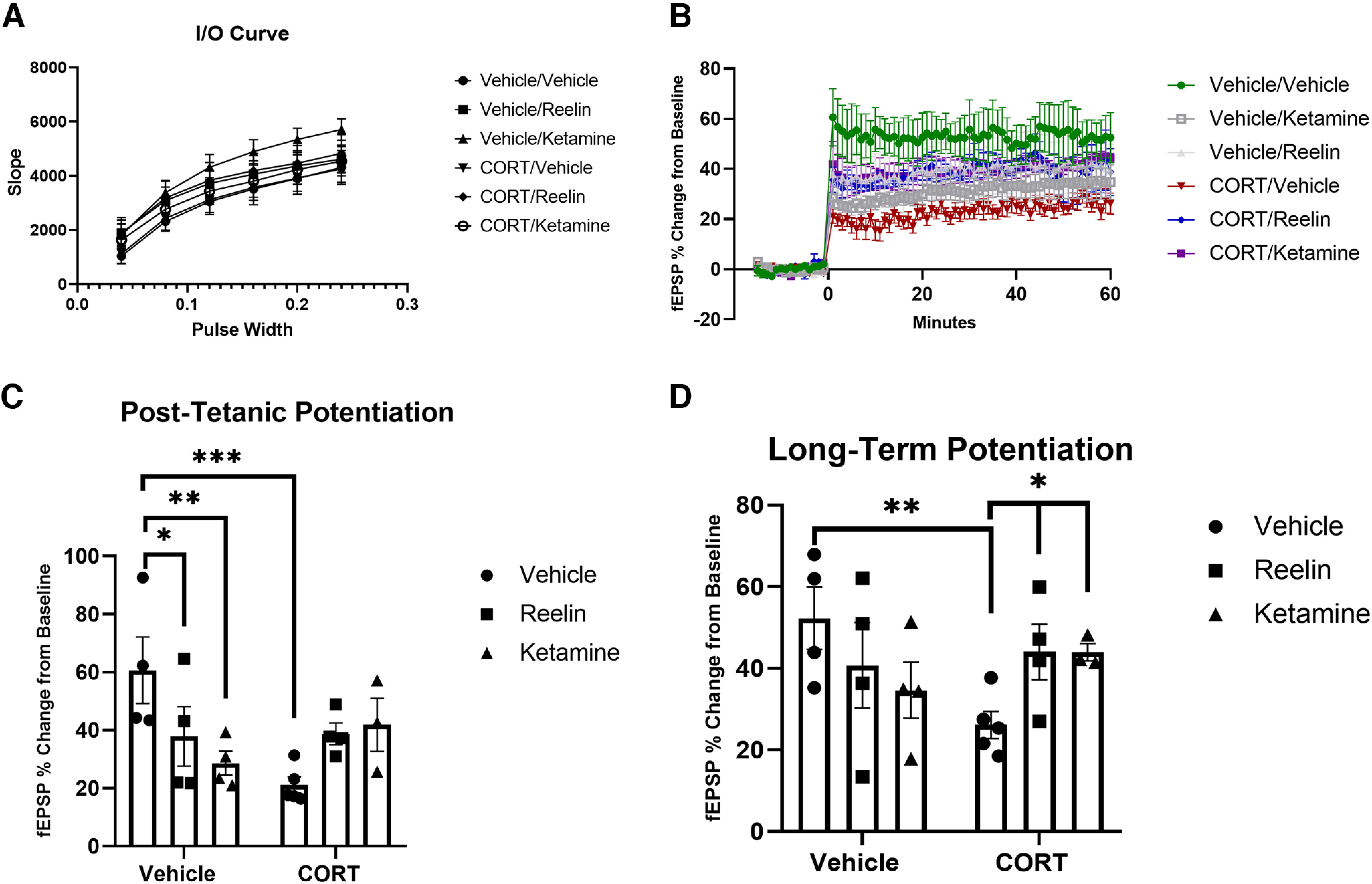
*In vivo* electrophysiological recordings from vehicle, CORT, reelin, and ketamine administered rats. ***A***, I/O function was measured using increasing pulse widths to determine baseline excitability. No differences were found between treatment groups. ***B***, Average fEPSP slope as a % change from baseline for vehicle/vehicle, CORT/vehicle, CORT/reelin, and CORT/ketamine experimental groups. A stable baseline was recorded for 30 min. θ Burst stimulation (TBS) was applied at 0 min. Post-tetanic potentiation (PTP) was measured at 1 min after θ burst stimulation (TBS) and long-term potentiation (LTP) was measured 55–60 min after TBS. ***C***, Changes in PTP potentiation after TBS. CORT significantly decreased PTP, which was not rescued by reelin or ketamine. ***D***, Changes in LTP after TBS. CORT administration significantly decreased LTP, which was rescued by both reelin and ketamine. All data expressed as mean ± SEM; *p < 0.05, **p < 0.01, ***p < 0.001.

Increasing pulse widths (0.04, 0.08, 0.12, 0.16, 0.2, and 0.24 mA) were measured before baseline measurements and after a stable signal was found to determine input/output (I/O) function. No significant differences were found in fEPSP slopes between any experimental subgroups, suggesting baseline synaptic excitability was similar. In all animals, the fEPSP slope was significantly greater with increased stimulation (Fig. 4*A*).

#### PTP and LTP

After a stable baseline was collected for a minimum of 30 min, the TBS protocol was used. Following TBS, the baseline stimulation protocol was continued for 1 h, with PTP defined as 1 min after TBS and LTP as 55–60 min after TBS. Mean traces of vehicle/vehicle, CORT/vehicle, CORT/reelin, and CORT/ketamine are graphed (Fig. 4*B*). Analyses were conducted on all 6 groups through a two-way ANOVA followed by Tukey’s *post hoc* tests whether the initial ANOVA was significant. Significant group differences were found for PTP (*F*_(2,18)_ = 7.179, *p* = 0.0051, η^2^ = 0.47) and LTP (*F*_(2,18)_ = 3.924, *p* = 0.0385, η^2^ = 0.32). The CORT/vehicle subgroup demonstrated significantly lower PTP than vehicles (*p* = 0.0008), although this was not significantly rescued by reelin or ketamine (Fig. 4*C*). Interestingly, PTP in vehicle animals was decreased by both reelin (0.0415) and ketamine (0.0064). LTP was significantly decreased after CORT treatment (*p* = 0.0045) but was rescued by both reelin (*p* = 0.0342) and ketamine (*p* = 0.0493; Fig. 4*D*).

### Reelin immunoreactivity in the subgranular zone

**Figure 5.**
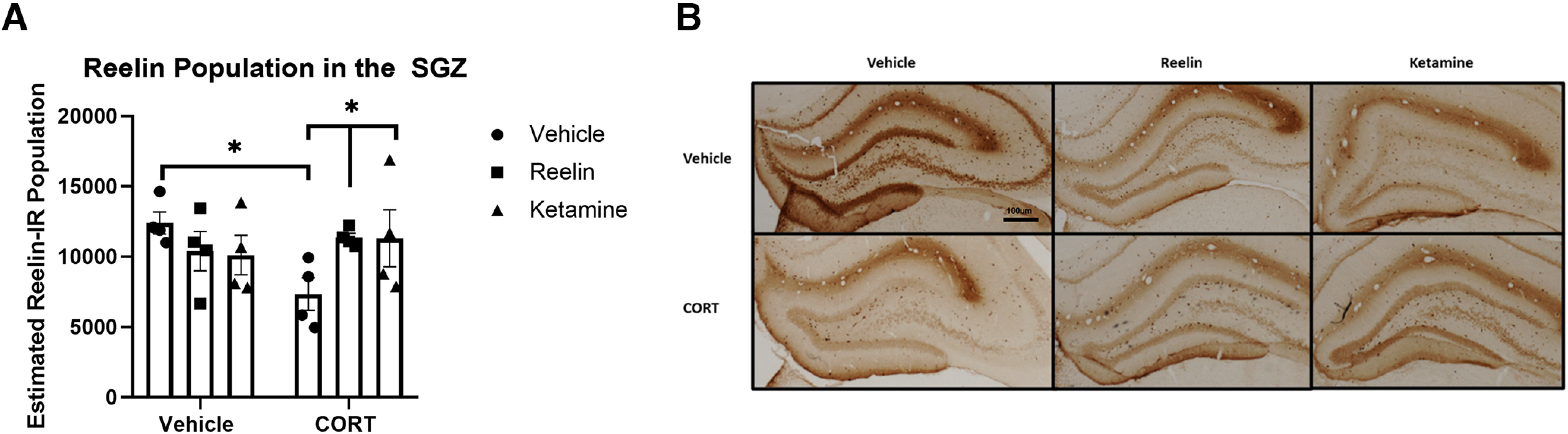
Reelin expression in the subgranular zone after reelin and ketamine administration. ***A***, Unbiased stereology estimates of reelin cell population in the dentate gyrus subgranular zone. CORT administration significantly decreased reelin expression, which was rescued by an acute dose of reelin and ketamine. ***B***, Representative photomicrographs of reelin expression in the subgranular zone. All data expressed as mean ± SEM; *p < 0.05.

Early research demonstrated that reelin and ketamine can increase reelin immunoreactivity in the subgranular zone of the hippocampus within a few days (Brymer et al., 2020; Johnston et al., 2020; Allen et al., 2022). However, no assessments have determined whether this effect is observed within 24 h and may be associated with the rapid-acting behavioral effects observed with each therapeutic. Significant condition and treatment differences were found in reelin immunoreactivity (*F*_(2,18)_ = 3.732, *p* = 0.0441, η^2^ = 0.33). Chronic CORT treatment significantly decreased the expression of reelin in the subgranular zone of the hippocampus (*p* = 0.0129), which was upregulated within 24 h by peripheral administration of both reelin (*p* = 0.0421) and ketamine (*p* = 0.0443; Fig. 5*A*,*B*).

### Western blot analyses

**Figure 6.**
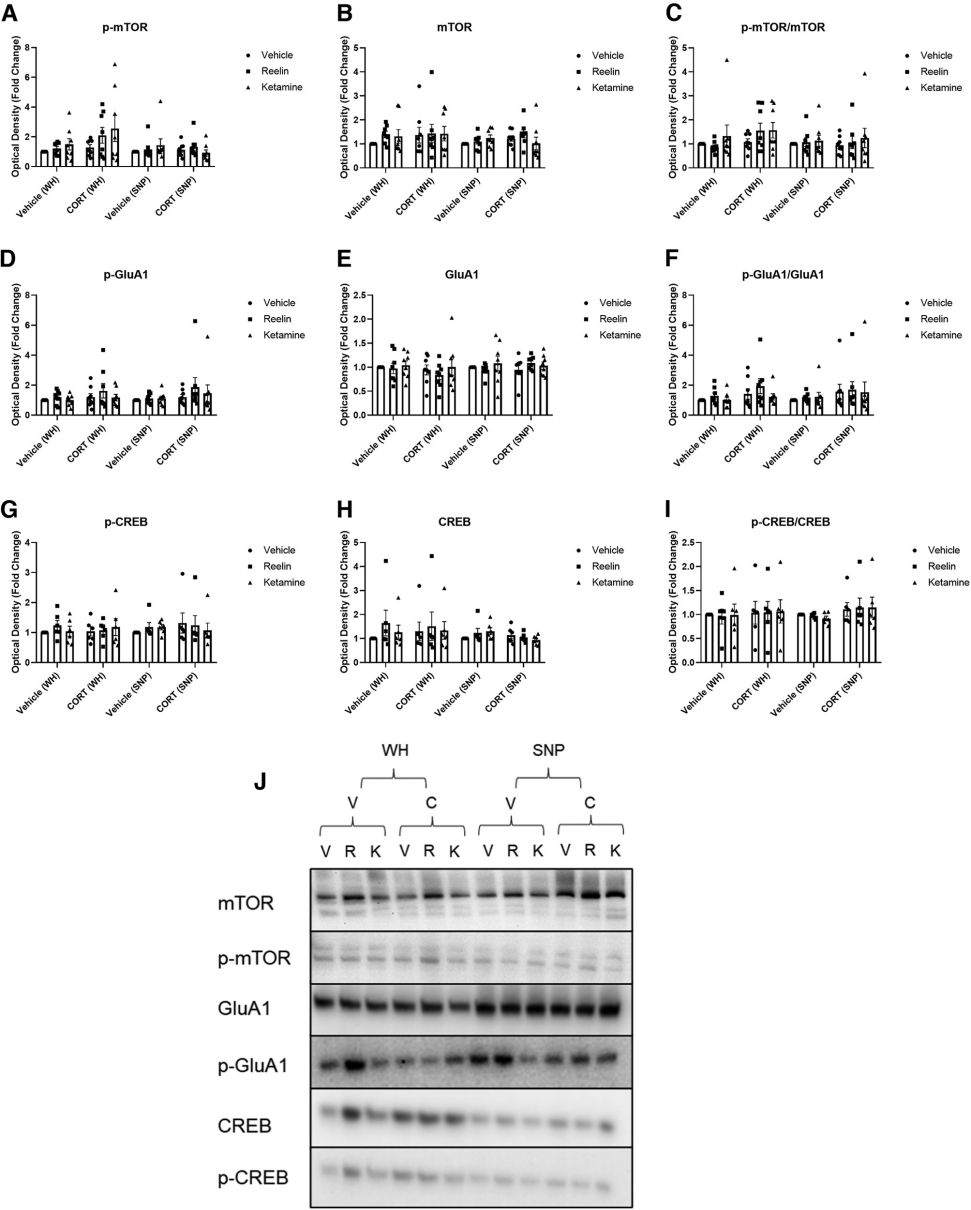
The effect of CORT, reelin, and ketamine treatment on whole cell and synaptic proteins (part I). ***A***, Whole cell p-mTOR expression was increased after ketamine administration. ***B–J***, No significant differences were found between any treatment groups for all proteins and activity ratios measured. V, vehicle; R, reelin; K, ketamine; C, CORT; WH, whole homogenate; SNP, synaptoneurosomes. All data are expressed as mean ± SEM; *p < 0.05.

**Figure 7.**
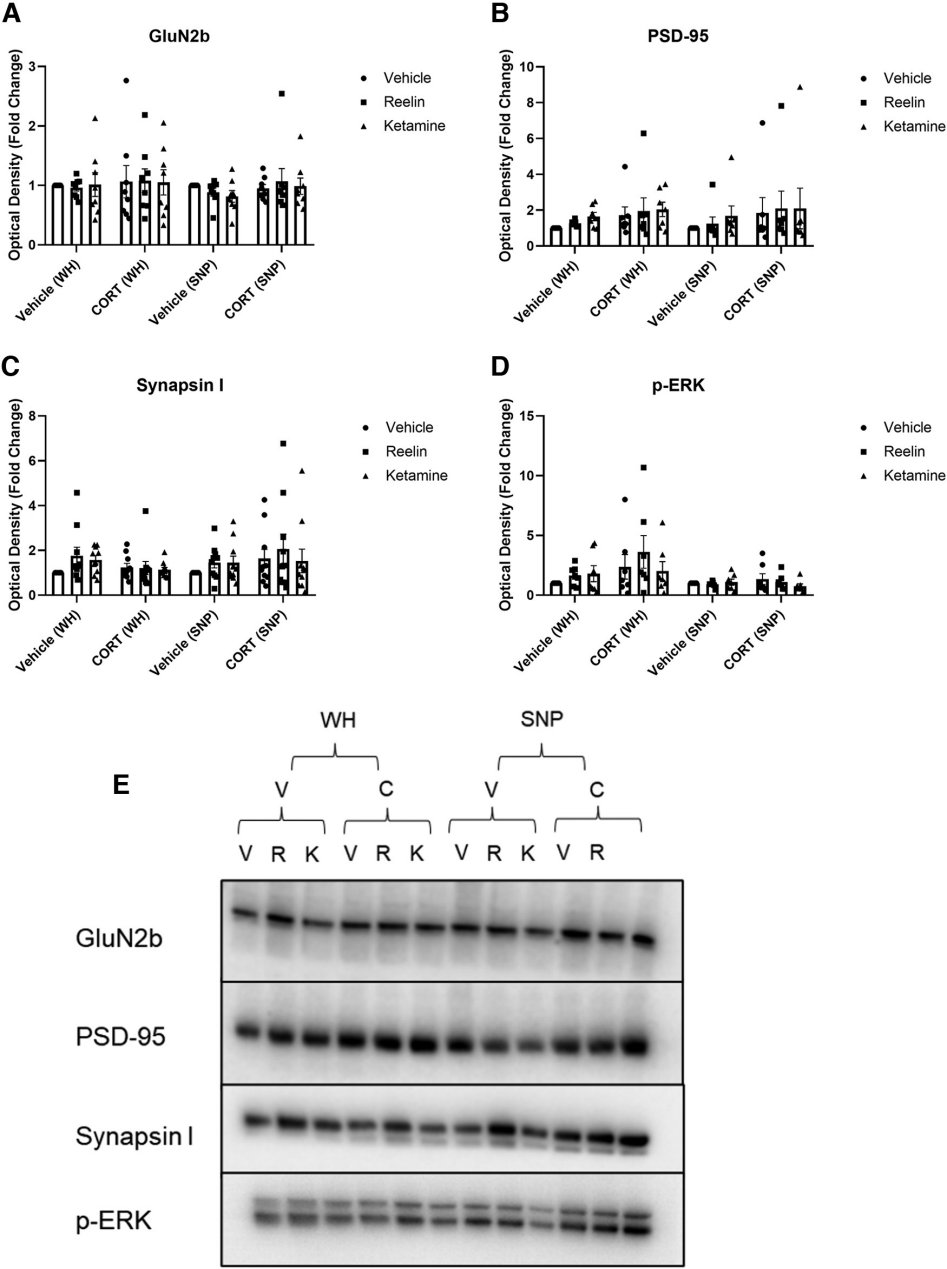
The effect of CORT, reelin, and ketamine treatment on whole cell and synaptic proteins (part II). ***A–E***, No significant differences were found between any treatment groups for all proteins measured. V, vehicle; R, reelin; K, ketamine; C, CORT; WH, whole homogenate; SNP, synaptoneurosomes. All data are expressed as mean ± SEM; *p < 0.05.

Western blotting analyses were conducted to evaluate condition and treatment differences in both whole homogenate (WH) and SNP tissue for GluN2b, PSD-95, Synapsin I, and p-ERK1/2, p-mTOR, mTOR, ratio of active mTOR, p-GluA1, GluA1, ratio of active GluA1, p-CREB, CREB, and ratio of active CREB. No significant effects of the condition or treatment were found for all proteins measured (Figs. 6, 7).

## Discussion

This exploratory study is the first to demonstrate that an acute peripheral dose of reelin is able to rescue behavioral, electrophysiological, and molecular deficits caused by chronic stress in a rapid-acting manner. These changes were found to be similar to those induced by ketamine, the current gold standard rapid-acting antidepressant. The dose of reelin used (3 µg) was found to be the most effective in rescuing behavioral and biological deficits in the CORT model, as demonstrated by previous research from our laboratory (Allen et al., 2022).

Behaviorally, reelin and ketamine rescued the increase in immobility and decrease in swimming induced by CORT in the FST, a proxy of despair-like behavior (Porsolt et al., 1978). Multiple previous studies have supported this finding after either reelin and ketamine administration (Fitzgerald et al., 2019; Brymer et al., 2020; Allen et al., 2022) and the FST is often used as an indicator of antidepressant efficacy in animal models (Kitada et al., 1981). Interestingly, the latency to immobility was rescued solely by reelin, which suggests animals receiving reelin may have improved performance relative to those receiving ketamine. The CORT-treated rats displayed greater anhedonic-like behavior than controls, however this was not significantly rescued by ketamine or reelin in this model. However, the vehicle animals who received ketamine also had significantly decreased sucrose consumption, a finding which parallels clinical trials in healthy volunteers who display greater depressive behaviors after ketamine administration (Derntl et al., 2019; Nugent et al., 2019).

To our knowledge, this study was the first to demonstrate that reelin can rescue hippocampal LTP in a chronic stress model. As previously described, LTP is an essential process for learning and memory, which are known to be negatively affected in depression (Strömgren, 1977; Weingartner et al., 1981; Kizilbash et al., 2002). Context discrimination and fear learning in particular have been associated with medial perforant path activity (Ferbinteanu et al., 1999), both of which are impacted in patients with depression and in chronic stress models (Britton et al., 2011; Camp et al., 2012; Wurst et al., 2021). Cortical and hippocampal increases in excitatory transmission have also been implicated in the efficacy of ketamine as an antidepressant (Zanos et al., 2018b; Gilbert and Zarate, 2020), and the parallel actions of reelin in this study provide further support for its putative rapid-acting antidepressant-like actions. Interestingly, ketamine administration decreased PTP in the control group, a finding that parallels the clinical research which demonstrates the deleterious effects of ketamine in healthy controls (Derntl et al., 2019; Nugent et al., 2019), perhaps because of its dissociative side effects. Reelin also caused a slight decrease in PTP in controls, but this reduction was not at the same magnitude as ketamine. As reelin was administered peripherally, conclusions cannot be made about the direct electrophysiological actions of reelin in the hippocampus. However, because of the high weight of reelin, hippocampal tissue damage and low diffusion rates from direct administration could impact the quality of electrophysiological signals.

The molecular indicators of antidepressant-like efficacy demonstrated more mixed results. Chronic CORT administration decreased reelin immunoreactivity in the subgranular zone of the dentate gyrus, a finding paralleled by previous research (Lussier et al., 2011, 2013; Fenton et al., 2015; Lebedeva et al., 2020). The ability of either reelin or ketamine to rescue reelin expression has also been previously demonstrated (Johnston et al., 2020; Kim et al., 2021; Allen et al., 2022); however, these changes had not yet been noted within 24 h as shown in this study. Despite previous research showing changes in SNPs after reelin and ketamine administration (Li et al., 2010; Johnston et al., 2020), no significant changes were measured in this context. Of note, the previous changes observed in SNPs after *in vivo* administration of ketamine disappeared by ∼2 h postinjection (Li et al., 2010). Previous *in vitro* assessments of reelin found changes after 30 min (Johnston et al., 2020), suggesting the time course of this research may have missed synaptic-specific changes in response to rapid-acting therapeutics.

There are still many questions regarding how a peripherally administered dose of reelin can have rapid-acting antidepressant effects and provoke changes in the CNS within 24 h after administration. Previous research has found evidence of reelin in the caveolae of endothelial cells and associated with the luminal side of the plasma membrane (Perez-Costas et al., 2015). This evidence suggests that despite its large size (410 kDa), reelin may cross the blood-brain barrier (BBB) through a receptor-mediated transcytosis mechanism. In addition, leakage of the BBB has been reliably found in depression, where increased permeability may allow for the crossing of molecules that are unable to in healthy controls (Najjar et al., 2013; Dudek et al., 2020). Reelin signaling through Dab1 activity has also been identified as a modulator of the BBB, organizing neuro-glia-vessel communication to support BBB integrity, and prevent leakage of interleukin-6 after chronic stress (Segarra et al., 2018; Zhang et al., 2020).

After crossing the BBB, tight gap junctions, pericytes, and astrocytic endfeet would most likely prevent the diffusion of reelin. However, evidence has shown that a unilateral intrahippocampal infusion of reelin is able to rapidly rescue deficits in both hemispheres, suggesting the activation of cellular signaling pathways can reverse deficits throughout the brain (Brymer et al., 2020). This could potentially be mediated through an increase in AMPAR throughput, as demonstrated by previous research which ascertained the administration of NBQX blocks therapeutic effects after an intrahippocampal infusion of reelin. While not observed here, increases in mTORC1 and ERK signaling have been observed after reelin administration *in vitro*, and warrant further investigation (Brymer et al., 2020; Johnston, J.N., Caruncho, H.J., and Kalynchuk, L.E., unpublished data). Future research should ascertain the exact mechanisms by which peripheral reelin is able to rescue behavioral and electrophysiological deficits through the blockade of AMPAR and NMDAR throughput, as well as mTORC1 and ERK signaling, which have all been associated with ketamine’s rapid-acting antidepressant actions (Zanos and Gould, 2018).

In addition, the peripheral mechanisms by which reelin could influence central protein translation are yet unknown. Reelin is expressed in blood plasma through platelet secretion, which has been associated with hemostasis and inflammatory responses (Tseng et al., 2014). Reelin has also been demonstrated to alter the membrane protein clustering (MPC) of the serotonin transporter (SERT) both *in vitro* and *in vivo* (Johnston et al., 2020; Allen et al., 2022). SERT is a primary target of conventional antidepressants, and parallel changes in SERT MPC have been observed in the chronic CORT model and treatment-naive participants with depression (Romay-Tallon et al., 2018). Reelin may also have its rapid-antidepressant-like actions through crossing the BBB and/or a reduction in the inflammatory response.

This study aimed to present a broad exploration of the changes induced in behavioral, electrophysiological, and molecular measures 24 h after acute peripheral reelin administration. Ketamine was used as an active comparator, as it is the current gold standard in rapid-acting antidepressant treatment. Given the exploratory nature of this study, small sample sizes may have limited statistical power that prevented the observation of subtle differences. Additionally, while chronic CORT administration is a reliable and validated model for the study of depression (Gregus et al., 2005; Johnson et al., 2006; Gourley and Taylor, 2009; Marks et al., 2009; Sterner and Kalynchuk, 2010), animal models are unable to completely mimic every facet of clinical depression which limits translation (McKinney and Bunney, 1969; Tkacs and Thompson, 2006). Treatment-resistant depression, the most common indication for use of rapid-acting antidepressants, is particularly difficult to model (Willner and Belzung, 2015).

Future research needs to expand on these preliminary findings and use a broad spectrum of animal models to assess reelin’s putative rapid-acting effects. Attaining a homeostatic balance of reelin, which is known to be downregulated in depression and other neuropsychiatric disorders, is imperative. While this study administered a low dose of reelin to upregulate reelin signaling and ameliorate behavior associated with chronic stress, significant increases in reelin expression can be a risk factor for the development of certain cancers and hepatic fibrosis (Khialeeva and Carpenter, 2017). In addition, determining the time scale of reelin’s effects is essential, as ketamine is known to act within 1 h and up to two weeks after administration (Murrough et al., 2013; Kishimoto et al., 2016). To our knowledge, no research has ascertained the length of reelin’s effects in a chronic stress model. In conclusion, this work provides multiple perspectives on how reelin may act as a putative rapid-acting treatment for chronic stress in a similar manner to ketamine, the gold standard for clinical antidepressant treatment.

## Synthesis

Reviewing Editor: Michael Michaelides, NIDA-NIH Decisions are customarily a result of the Reviewing Editor and the peer reviewers coming together and discussing their recommendations until a consensus is reached. When revisions are invited, a fact-based synthesis statement explaining their decision and outlining what is needed to prepare a revision will be listed below. The following reviewer(s) agreed to reveal their identity: NONE. Note: If this manuscript was transferred from JNeurosci and a decision was made to accept the manuscript without peer review, a brief statement to this effect will instead be what is listed below.

Reviewer 1

The authors did not perform any of the experiments requested. However, overall the manuscript has been substantially improved, thus I feel that this body of work deserves publication.

Reviewer 2

Johnston et al. addressed all of my comments well.

## Author Response

Reviewer 1

The manuscript by Johnston et al shows that a peripheral dose of reelin rapidly ameliorates behavioral, electrophysiological, and molecular deficits observed in rats exposed to a chronic (21 days) regimen of CORT. Overall, the study is of interest to the field. However, the small sample size together with the use of forced swim test as behavioral construct to study antidepressant effects lower the appeal of the current work.

We thank the reviewer for their comments and have now included further behavioral tests in the manuscript (the sucrose preference test and splash test for grooming) alongside changes in framing from a “depressive-like” phenotype to a “stress/stress-coping” phenotype. Moreover, there is a clear disconnect from neuroadaptation observed electrophysiologically as well as molecularly in the hippocampus and the production of the behavioral effects driven by reelin. We hope the changes made regarding the major and minor criticisms of the reviewer help increase the understanding of a potential connection between electrophysiological data and molecular measures in the hippocampus. Here below the authors will find my major criticisms:

- In order make all the hippocampus data of any relevance to the behavioral domain the authors should attempt to microinject reeling within the hippocampal formation and eventually observed rapid and sustained amelioration of stress-induced deficits at the molecular, electrophysiological, and behavioral level.

We agree completely, and refer the reviewer to the paper conducted by our lab previously

published:

Brymer, K. J., Johnston, J., Botterill, J. J., Romay-Tallon, R., Mitchell, M. A., Allen, J., ... &

Kalynchuk, L. E. (2020). Fast-acting antidepressant-like effects of Reelin evaluated in the

repeated-corticosterone chronic stress paradigm. Neuropsychopharmacology, 45(10), 1707-1716.

In this paper, we found that microinjections of reelin (both repeated and acute) rescued stress-induced deficits in object-location memory, forced-swim test immobility, AMPA, NMDA, and GABAa receptors. Repeated reelin microinjections into the hippocampus was also able to rescue the number and complexity of newborn neurons as determined by DCX+. Electrophysiology was not conducted in this study, as a microinjection of reelin (∼388 kDa) directly into the hippocampus impacts hippocampal structure and would impact short-term excitatory signaling that we hoped to capture with our in vivo electrophysiology data in this study. For the purpose of this study, we also hoped to elaborate on this direct microinjection work and determine whether peripheral injections of reelin (a more translatable model) could have rapid acting effects. While we hope to assess more sustained effects in the future, we believe this may

be out of the scope of the current study.

In the text, we have made the conclusions from the previous experiments more clear, as well as justification for not conducting in vivo electrophysiology after direct hippocampal reelin microinjections as follows:

In the introduction: “Previous research from our laboratory has demonstrated that both repeated and acute intrahippocampal infusions of recombinant reelin (1 µg) were able to rescue stress-induced deficits in object-location memory, forced-swim test immobility, and hippocampal AMPAR, NMDAR, and GABAAR expression in the hippocampus (Brymer et al., 2020). In addition, peripheral administration of reelin through the lateral tail vein demonstrated a parallel rescue of behavioural and biological chronic stress phenotype (Allen, 2022).” In the discussion: “As reelin was administered peripherally, conclusions cannot be made about the direct electrophysiological actions of reelin in the hippocampus. However, due to the high weight of reelin, hippocampal tissue damage and low diffusion rates from direct administration could impact the quality of electrophysiological signals.”

- The observation that Reelin is capable of rescuing synaptic plasticity within the hippocampus is of potential relevance. However, all the electrophysiological experiments have a very low sample size. While we agree that sample sizes are quite low given the exploratory nature of this study, power analysis for the electrophysiology experiments was conducted based on a pilot conducted in our lab, which determined an n of 4 in each experimental group would be suitable to determine significance. However, we have now also reported effect sizes for each experiment and softened the language to reflect this.

Moreover, there is clearly a missing experiment in this current body of work. That is, preventing AMPAR-mediated synaptic transmission in vivo (selectively in the hippocampus) while animals are experiencing systemic delivery of reelin should prevent reelin mediated antidepressant effects. Without such experiments all the claims are merely correlative. Similarly, previous work from our laboratory has demonstrated that utilizing CNQX to block AMPAR transmission in the hippocampus blocked the rescue of the behavioral phenotype

observed after chronic corticosterone injections:

Brymer, K. J., Johnston, J., Botterill, J. J., Romay-Tallon, R., Mitchell, M. A., Allen, J., ... &

Kalynchuk, L. E. (2020). Fast-acting antidepressant-like effects of Reelin evaluated in the repeated-corticosterone chronic stress paradigm. Neuropsychopharmacology, 45(10), 1707-1716. We agree it would be extremely interesting to determine if blocking AMPAR transmission in the hippocampus would affect in vivo electrophysiological measures, however, have similar concerns as mentioned above regarding how microinjecting reelin could affect the hippocampal

structure and signaling. We have also included further details in the text as follows:

In the introduction: “The actions of reelin in chronic stress models may be activated through similar AMPAR-mediated downstream cellular signaling pathways to ketamine, as blocking AMPAR signaling with CNQX prevents reelin's ability to rescue a chronic stress phenotype (Brymer et al., 2020). Additionally, blockade of reelin’s receptor apolipoprotein E receptor 2 and related downstream Src family signaling kinases prevented ketamine’s actions in a

depressive-like phenotype (Kim et al., 2021).”

In the discussion: “As reelin was administered peripherally, conclusions cannot be made about the direct electrophysiological actions of reelin in the hippocampus. However, due to the high weight of reelin, hippocampal tissue damage and low diffusion rates from direct administration could impact the quality of electrophysiological signals.”

Minor criticisms:

-The title of the manuscript does not reflect the body of work presented by the authors. -In the manuscript the authors should try to avoid the language “depressive-like behavior” and substitute this construct with passive coping, a more accurate term in this context. We have now changed the title to better reflect the phenotype we are measuring, changing mentions in the text and title from “depressive-like” to “stress-coping” or “chronic stress phenotype”.

-Plots in all the Figures are way too small and very difficult to read. Figures have been improved and uploaded separately in the resubmission in order to improve image quality.

Reviewer 2

Johnston et al attempt to compare systemic treatment with the extracellular matrix protein reelin to ketamine after chronic treatment with corticosterone in rats. They compare forced swim test behavior, electrophysiological measures, and molecular effects of these two drugs after chronic stress. They find that reelin decreases immobility time in the FST, rescues reelin immunoreactivity in the hippocampus, and rescues LTP similarly to ketamine. They find few changes in protein expression; however, this could be due to the timepoint chosen. This research is quite worthwhile as depression is one of the most common psychiatric ailments and there are very few rapid-acting antidepressants.

The FST is a predictive test for antidepressant efficacy. However, the effects even for SSRIs, which take weeks to relieve depressive symptoms, are seen within 24 hours of administration. Thus, FST does not tell us whether the drug is a rapid-acting antidepressant or a more traditional antidepressant. The other recent papers from this group used the open-field test and the object-in place recognition test, which measure more anxiety-like and cognition/memory, respectively, than depression-like behavior. Thus, without testing reelin in another modality like sucrose preference test or emergence test, it is difficult to determine if reelin is indeed a rapid-acting antidepressant, so even calling it a “putative rapid-acting antidepressant” seems premature. We completely agree and have included the Sucrose Preference Test data, an indicator of anhedonic-like behaviour, conducted with this experiment to better inform the reader of the effects of reelin in the chronic stress model. Additionally, we have softened the language throughout the manuscript to “stress-coping” or chronic stress phenotype in order to better reflect the model used.

Minorly, figure 2 is not referred to in the text, which clearly needs to be resolved.

Figure 1 is slightly confusing as it seems to indicate all rats underwent the FST, whereas the manuscript indicates that the electrophysiology rats did not. Apologies, we have corrected these two mistakes. Figure 2 is now referred to in the text as

follows:

"In the forced swim test, significant differences were found in measures of swimming [F (2, 41)

= 6.384, p = 0.0039, η2 = 0.34], immobility [F (2, 41) = 5.184, p = 0.0098, η2 = 0.35], and latency to immobility [F (1, 41) = 7.670, p = 0.0084, η2 = 0.23] (Fig. 2A - D). Post-hoc analyses revealed that the CORT/vehicle group spent significantly more time immobile (p = 0.0003), an increase that was rescued by reelin (p = 0.0049) and ketamine (p = 0.0047) (Fig. 2C). In parallel to this, swimming time was decreased in rats treated with CORT/vehicle (p = 0.0002), which was upregulated by reelin (p = 0.0028) and ketamine (p = 0.0034) (Fig. 2B). Finally, those in the CORT/vehicle subgroup had significantly shorter latency to immobility times when compared to vehicle-treated animals, which were rescued by reelin (p = 0.0423), but not ketamine (Fig. 2D). No effects on climbing behaviour were found (Fig. 2A).” And Figure 2 has been adapted to better reflect behavioral which experimental subgroups underwent behavioral testing, as well as to indicate the other behavioral tests conducted. In line 298, the authors state that ketamine appeared to decrease LTP in the control group, however, the statistics for this are not shown. In fact, the reelin and ketamine groups of control rats are never compared to anything which makes one wonder why those groups exist. Our apologies, the statistics were run to include the vehicle groups however were not reported. We have now included the post-hoc analyses between vehicle groups where applicable. The authors note that this is a preliminary and exploratory study, however, given the small group sizes (except for the FST), some of the claims made are worded too strongly and need to be toned down.

We have now toned down the language and conclusions throughout the manuscript, including emphasizing chronic stress rather than antidepressant-like actions.

Overall there is promise to this study, but it seems a little early to be published. We hope that with the inclusion of further behavioral experiments helps to increase the value of this study for publication.
